# miR-193a-3p regulates the multi-drug resistance of bladder cancer by targeting the LOXL4 gene and the Oxidative Stress pathway

**DOI:** 10.1186/1476-4598-13-234

**Published:** 2014-10-14

**Authors:** Hui Deng, Lei Lv, Yang Li, Cheng Zhang, Fang Meng, Youguang Pu, Jun Xiao, Liting Qian, Weidong Zhao, Qi Liu, Daming Zhang, Yingwei Wang, Hongyu Zhang, Yinghua He, Jingde Zhu

**Affiliations:** Cancer Epigenetics Program, Anhui Cancer Hospital, Hefei, 230031 Anhui China; Department of Biology, School of Life Science, Anhui Medical University, Hefei, 230031 Anhui China; Department of Urology, The First Affiliated Hospital of Harbin Medical University, Harbin, Heilongjiang Province China; Department of Radiotherapy, Anhui Cancer Hospital, Hefei, 230031 Anhui China; Department of Urology, Anhui provincial hospital, Hefei, China; Department of Gynecologic Cancer, Anhui Cancer Hospital, Hefei, 230031 Anhui China; School of Life Science and Technology, State Key Laboratory of Urban Water Resource and Environment, Harbin Institute of Technology, Harbin, 150001 Heilongjiang China; Department of Neurosurgery, The First Affiliated Hospital of Harbin Medical University, Harbin, Heilongjiang Province China; Department of Pathology, The First Affiliated Hospital of Harbin Medical University, Harbin, Heilongjiang Province China; Cancer Epigenetics Program, Shanghai Cancer Institute, Renji Hospital, Shanghai Jiaotong University, Shanghai, 200032 China

**Keywords:** LOXL4, miR-193a-3p, Chemoresistance, Bladder cancer, Oxidative stress pathway

## Abstract

**Background:**

Chemoresistance is a major obstacle to the curative cancer chemotherapy and presents one of the most formidable challenges in both research and management of cancer.

**Results:**

From the detailed studies of a multi-chemosensitive (5637) versus a chemoresistant (H-bc) bladder cancer cell lines, we showed that miR-193a-3p [GenBank: NR_029710.1] promotes the multi-chemoresistance of bladder cancer cells. We further demonstrated that lysyl oxidase-like 4 (LOXL4) gene [GenBank: NM_032211.6] is a direct target of miR-193a-3p and executes the former’s impact on bladder cancer chemoresistance. The Oxidative Stress pathway activity is drastically affected by a forced reversal of miR-193a-3p or LOXL4 levels in cell and may act at the downstream of LOXL4 gene to relay the miR-193a-3p’s impact on the multi-chemoresistance in both cultured cells and the tumor xenografts in nude mice.

**Conclusions:**

In addition to a new mechanistic insight, our results provide a set of the essential genes in this newly identified miR-193a-3p/LOXL4/Oxidative Stress axis as the diagnostic targets for a guided anti-bladder cancer chemotherapy.

**Electronic supplementary material:**

The online version of this article (doi:10.1186/1476-4598-13-234) contains supplementary material, which is available to authorized users.

## Background

Bladder cancer (BCa) is the second most frequent type of cancer in male around the world [1]. It is highly refractory to the drug therapy, frequently recurs in a more aggressive form after the initial surgical eradication, and therefore regarded as one of the most costly and difficult types of cancer to be contained [2]. Chemoresistance prevails in cancer clinic and is hard to be predicted in advance, preventing the effective cancer chemotherapy [3]. Despite years of intensive efforts, our mechanistic understanding of cancer chemoresistance remains elusive.

MicroRNAs (miRs) are a group of RNA species being around 22 nucleotides length and regulate expression of the protein coding genes in all physiological conditions of higher eukaryotes *via* its repressing effect at both translability and stability levels of mRNAs in a sequence specific fashion [4]. Among more than 2,000 known human miRs, several dozens are aberrantly expressed in cancer and have the proven roles in both initiation and progression of cancer [5]. The miRs upregulated in cancer cells often have the oncogenic role, and the well-known examples of this class are miR-10b, miR-17-92, miR-122 and miR-155 [6]. Conversely, there are miRs having the negative role in regulation of cell proliferation and are often down-regulated in cancer cells, such as let-7c, miR-10b, miR-15a, miR-31, miR-34, miR-145, miR-223 [7]. The miR’s prospects as the drug-specific target [8] and biomarker [9] have gained a great attention in recent years. The panel of miRs implicated in the initiation and maintenance of the cancer chemoresistance has also been reported [10], and the noticeable examples in BCa’s chemoresistance include miR-30d, miR-181, and miR-199a-5p [11].

The first indication that miR-193a-3p might be involved in tumor suppression was the observation that it was silenced by DNA methylation during oral carcinogenesis [12]. Dysregulation of miR-193a-3p was also reported in other types of cancer, such as non-small cell lung cancer (NSCLC) [13], prostate cancer [14], breast cancer [15], Head and Neck Squamous Cell Carcinomas [16], and colorectal cancer [17]. The carcinogenic impact of miR-193a-3p has been attributed to its repression of c-Kit [18] and the PTEN/PI3K signaling pathway in acute myeloid leukemia [19], of KRAS and PLAU in colon cancer [20], of PLAU [21] and EGFR-driven cell-cycle network proteins [22] in breast cancer, of ARHGAP19, CCND1, ERBB4, KRAS and Mcl-1 in epithelial ovarian cancer [23], of PLAU in hepatocellular carcinoma (HCC) [24], and of Mcl-1 in NSCLC [25]. MiR-193a-3p also induces an accumulation of intracellular reactive oxygen species (ROS) and DNA damage in cancer cells *via* targeting Mcl-1 [26]. It was recently reported to suppress NSCLC metastasis through downregulation of the ERBB4/PIK3R3/mTOR/S6K2 signaling pathway [27]. In contrast, several studies reported an oncogenic role for miR-193a-3p. For example, miR-193a-3p was shown to promote *in vivo* tumorigenesis of metastatic medullary thyroid carcinoma [28], and to enhance both tumor growth in nude mice and chemoresistance of HCC by targeting of the SRSF2 gene [29].

We show here that miR-193a-3p promotes the BCa multi-drug resistance phenotype *via* its repression of the lysyl oxidase-like 4 (LOXL4) gene, a newly identified direct target of miR-193a-3p. The LOXL4 protein is an important member of the lysyl oxidase (an extracellular copper-dependent amine oxidase) family that catalyzes the first step of the crosslinks between collagens and elastin during the biogenesis of connective tissue and is frequently deregulated in cancer. Mutations in the coding sequences of LOXL4 gene have also been reported in various types of cancer from the cancer genomic studies: **COSMIC** [http://cancer.sanger.ac.uk]. We also show here that the Oxidative stress (OS) pathway is the predominant pathway affected by miR-193a-3p *via* its repression of LOXL4 expression.

## Results

### The miR-193a-3p level was higher in the chemoresistant (H-bc and UM-UC-3) than the chemosensitive (5637) BCa cell lines

**Figure 1:**
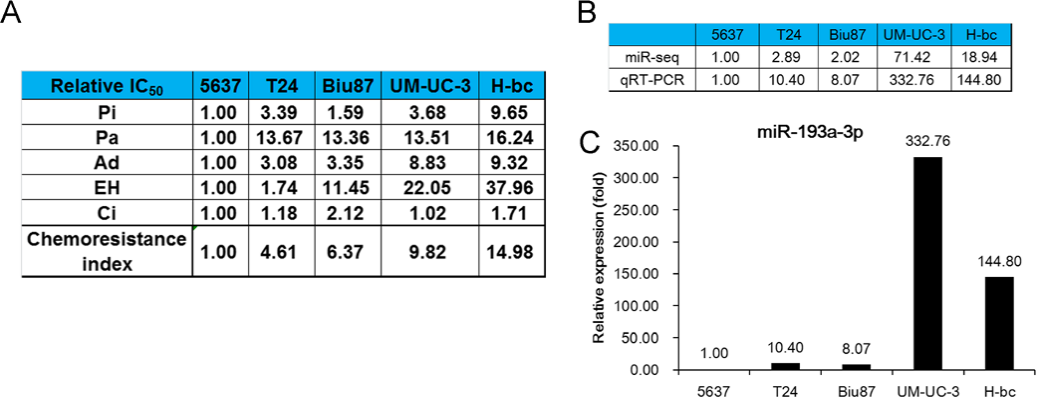
**The miR-193a-3p expression differs in the chemoresistance distinct bladder cancer cell lines.** Relative IC_50_ values (fold) of BCa cell lines to five chemotherapeutics with the lowest IC_50_ (5637 cell line) as a reference **(A)**. The relative miR-193a-3p level (fold) in BCa cell lines by both miR-seq and qRT-PCR analyses is shown in Table **(B)** and by qRT-PCR in plot **(C)**.

The dose required for 50% cells killed (IC_50_) after a 72 hours drug treatment by Pirarubicin(Pi), Paclitaxel(Pa), Adriamycin(Ad), Cisplatin(Ci) or Epirubicin Hydrochloride(EH) were determined in the following five BCa cell lines: 5637, T24, Biu87, H-bc and UM-UC-3. Judged by the fold difference over the lowest IC_50_, 5637 was the most multi-chemosensitive, while H-bc and UM-UC-3 were the most resistant cell lines (Figure 1A). Revealed by both a sequencing based miRomic analysis (not shown) and the qRT-PCR validation, the miR-193a-3p level was over 100 folds higher in both H-bc and UM-UC-3 than in 5637 cells (Figure 1B and C). All these observations suggest that miR-193a-3p may have a promoting role in the BCa chemoresistance as previously reported in the 5-FU resistance of HCC [29].

### LOXL4 mRNA is a direct target of miR-193a-3p in BCa cells

**Figure 2:**
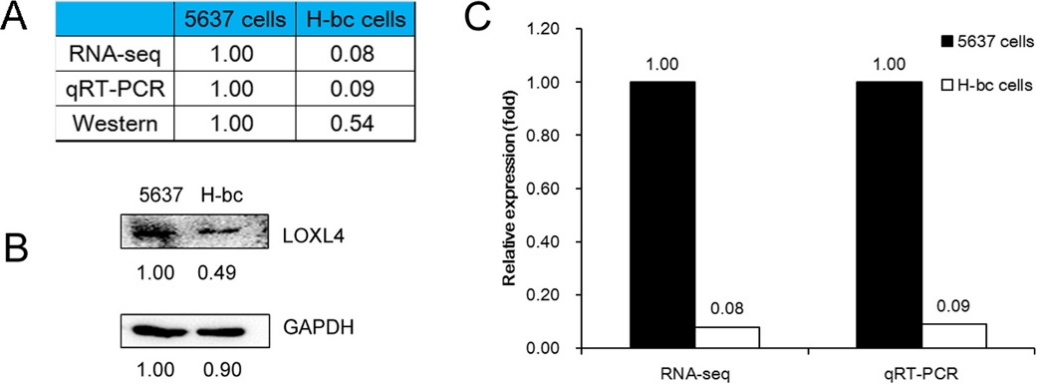
**The LOXL4 level is higher in 5637 than in H-bc cells.** The relative level (fold) of the LOXL4 gene in 5637 versus H-bc cells summarized in table **(A)**, analyzed by Western analysis **(B)**, by miR-seq and qRT-PCR analyses in plot **(C)**.

Any a given microRNA may regulate the expression of up to several hundred genes at the post-transcriptional level in both cellular content-dependent and sequence-specific manners. Besides SRSF2 [29], miR-193a-3p’s influence on the cancer chemoresistance is expected to be accomplished *via* repression of its other targets. To this end, we checked the level of 359 Targetscan (http://www.targetscan.org/)-predicted genes in the RNA-seq datasets (the RNA-seq omic data, not shown) of 5637, UM-UC-3 and H-bc cells. LOXL4 is one of several dozen genes that differentially expresses in a pattern opposite to the miR-193a-3p’s. Further qRT-PCR and Western analyses showed that LOXL4 level is significantly higher in 5637 than in H-bc cells at both mRNA (RNA-seq based omic analysis: 1.00:0.08, and the qRT-PCR analysis: 1.00:0.09) and protein levels (Western analysis: 1.00:0.54, Figure 2). The LOXL4 expression in another multi-chemoresistant cell line, UM-UC-3 cells was at an undetectable level (not shown).

**Figure 3:**
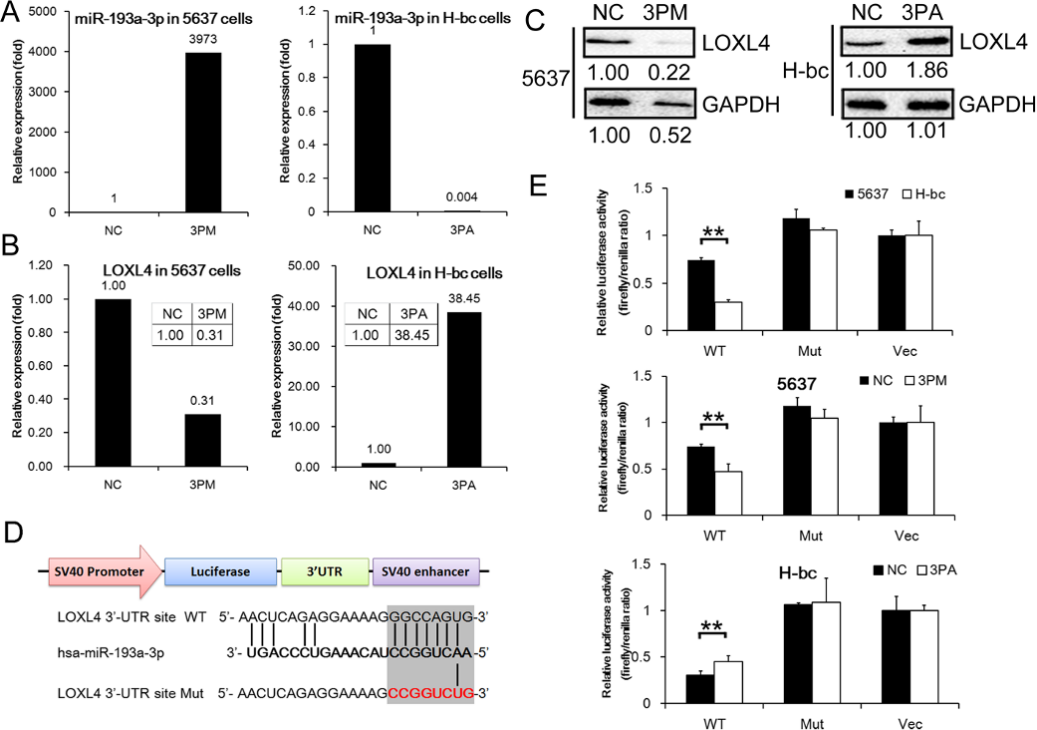
**The LOXL4 is a direct target of miR-193a-3p in BCa cells.** The level of miR-193a-3p **(A)**, the LOXL4 mRNA l **(B)** and protein **(C)** in the miR-193a-3p mimic (3 PM) transfected 5637 cells and the miR-193a-3p antagomiR (3PA) transfected H-bc cells versus the negative control (NC), determined by qRT-PCR or Western analyses. **D**, The sequences (the wild-type in shadow and mutant in red) in UTR region of LOXL4 gene targeted by miR-193a-3p. **E**, The relative luciferase activity (fold) of the reporter with wild-type (WT) or mutant (Mut) LOXL4-UTR or with no UTR (Vec) were determined in the miR-193a-3p mimic (in 5637) or antagomir (in H-bc) or Mock transfected BCa cells. The Renilla luciferase activity of a co-transfected control plasmid was used to control the transfection efficacy. The representative results from three independent experiments shown. Error bars represent s.e.m. **P < 0.01; by Student’s *t*-test.

We further determined the LOXL4 level in both miR-193a-3p mimic transfected 5637 and the antagomiR transfected H-bc cells versus the mock transfected. In parallel with the changes of the miR-193a-3p level (Figure 3A), a miR-193a-3p mimic transfection brought down the LOXL4 mRNA level by nearly 70% (Figure 3B) and the protein level by 58% (Figure 3C) in 5637 cells. As expected, a miR-193a-3p antagomiR transfection raised the mRNA level of LOXL4 by over 37 folds (Figure 3B) and the protein level by 84% in H-bc cells (Figure 3C).

To conclude that LOXL4 gene is a direct target of miR-193a-3p, we put the wild type or mutant 3′-UTR region (1325 bp) at the downstream of the firefly luciferase gene of pGL3 vector (Promega) to create pGL3-LOXL4 UTR WT and the PGL3-LOXL4 UTR Mut, respectively (Figure 3D). Both constructs and pGL3 were transfected into 5637 and H-bc cells respectively, to determine whether the chemoresistance associated expression of miR-193a-3p in BCa cells is indeed functional. pGL3-LOXL4 UTR WT but not other two reporter constructs gave a significantly higher luciferase activity in 5637 than H-bc cells (Figure 3E). Furthermore, the luciferase activity of pGL3-LOXL4-UTR WT but not other two was brought down by the mimic in 5637 cells and raised by the antagomiR transfection in H-bc cells (Figure 3D). Getting all these together, LOXL4 is indeed, a direct target of miR-193a-3p and may execute the miR-193a-3p’s effect on the BCa chemoresistance.

### A siRNA mediated LOXL4 repression essentially reproduced the miR-193a-3p mimic’s effect on the chemoresistant state of 5637 cells

**Figure 4:**
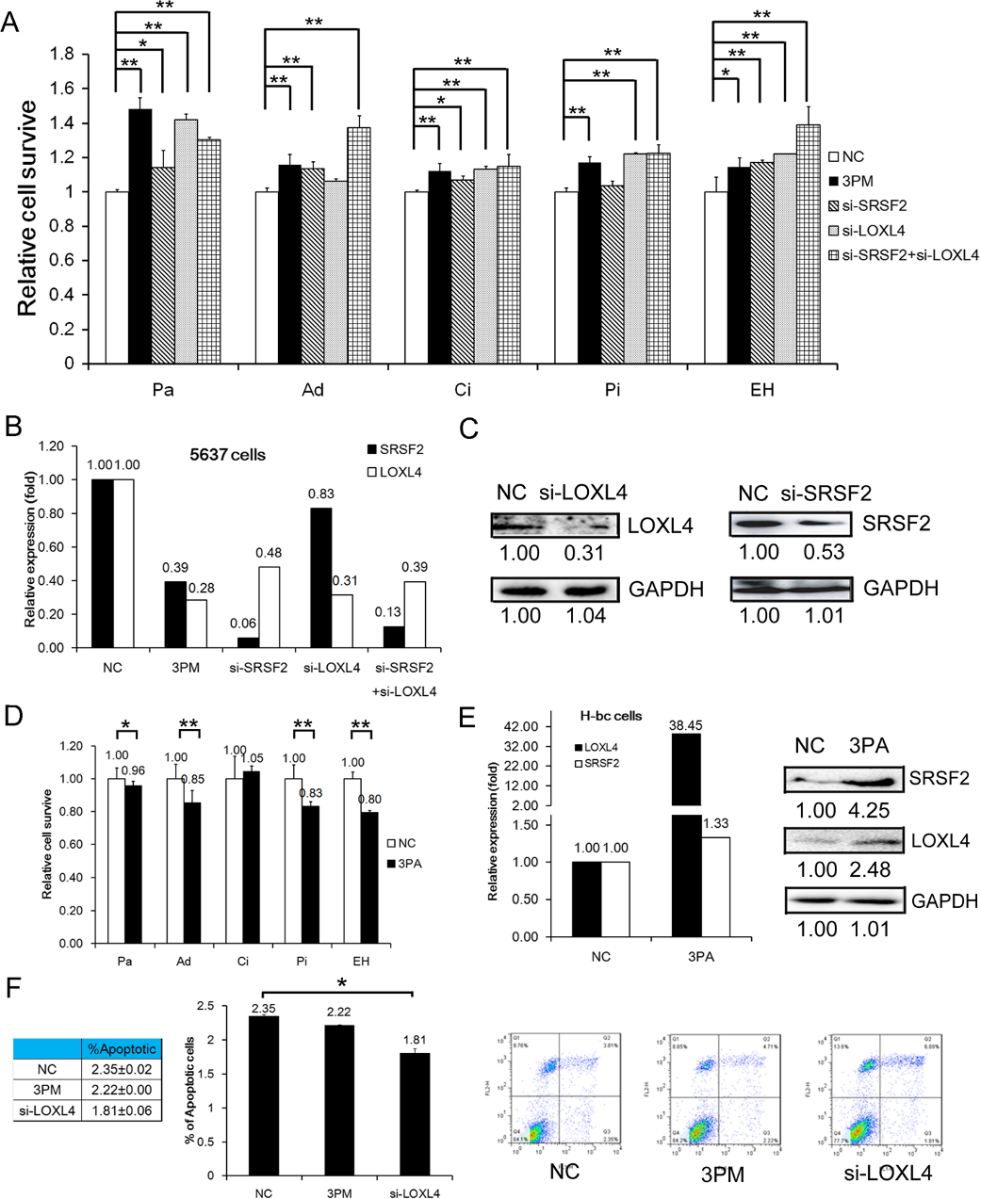
**The effects of a forced reversal of the miR-193a-3p or LOXL4 levels on the chemoresistance of 5637 and H-bc cells. A**, The IC_50_ dosed drug-triggered cell death of 5637 cells transfected by miR-193a-3p mimic (3 PM) or the gene specific siRNAs versus the negative control (NC). **B**, The levels by qRT-PCR of both LOXL4 and SRSF2 mRNAs in the 3 PM, or siRNAs (single or in combination) transfected 5637 cells and the 3PA transfected H-bc cells versus the NC. **C**, The levels by Western analysis of both LOXL4 and SRSF2 proteins in the siRNA versus the negative control siRNA (NC) transfected 5637 cells. **D**, The drug-triggered cell death of the miR-193a-3p antagomiR (3PA) versus the NC transfected H-bc cells. **E**, The levels of both LOXL4 and SRSF2 mRNAs and proteins were measured by qPCR and Western analyses in the 3PA versus NC transfected H-bc cells. **F**, The effects of the forced reversal of both miR-193a-3p and LOXL4 levels on the apoptosis by FACS analysis of 5637 cells in plot and in the original. (*,P < 0.05; **,P < 0.01).

To explore the LOXL4 role in the BCa chemoresistance, we transfected 5637 cells with the miR-193a-3p mimic and the siRNAs of SRSF2 and LOXL4, respectively, and assayed the cell death triggered by an IC_50_ dosed drug. The cell death triggered by all the five drugs was significantly reduced in the miR-193a-3p mimic transfected 5637 cells (Figure 4A). In parallel with the reduction of both target mRNA and protein levels (Figure 4B and C), the siRNA mediated LOXL4 repression reduced the cell death triggered by four of five drugs, excluding Ad, while the siRNA mediated SRSF2 repression relieved the cell death triggered by four drugs, excluding Pi instead (Figure 4A). These observations suggest that LOXL4 and SRSF2 have significantly overlapped but distinguished roles in execution of the miR-193a-3p’s impact on the BCa chemoresistance in a drug-specific fashion. Furthermore, an additive (or synergistic) effect on both EH and Ad triggered cell death, but not on the other drugs were revealed in 5637 cells co-transfected by both siRNAs (Figure 4A). Essentially as expected, a miR-193a-3p antagomiR transfection sensitized H-bc cells to the cell death triggered by four drugs, excluding Ci (Figure 4D). As a measure of the successful transfection, the level of both LOXL4 and SRSF2 mRNAs was altered in a predicted direction in antagomiR transfected cells (Figure 4E). In line with its negative effect on chemoresistance, a siRNA mediated LOXL4 repression lowered the percentage of apoptotic cells from 2.35% to 1.81%, an effect was not seen in the mimic transfected 5637 cells (Figure 4F). Despite this difference, LOXL4 indeed plays an essential part in the miR-193a-3p’s effect on the multi-chemoresistance of BCa cells, except for the Ad resistance.

It is surprising to find a significant discrepancy between the mRNA level (by qRT-PCR) and protein level (by Western blotting analysis) of SRSF2 and LOXL4 altered by miR-193-3p mimic or antagomiR, the alteration of the protein level is far limited than that of mRNA. It has been established that the relative contribution of a miR mediated translation repression and RNA degradation promotion to the expression of its target genes is target gene specific. Indeed, it has been shown that for highly repressed targets, mRNA destabilization usually comprised the major component of repression by miRs [30]. Relevant to this issue, Wu, L et al. have recently shown that miRs also serves as a surveillance system to repress the expression of nonsense mRNAs that may produce harmful truncated proteins [31]. It is thus very likely that the observed massive reduction/increase of SRSF2 and LOXL4 mRNA by the mimic (in 5637 cells)/antagomiR in H-bc cells reflects mainly the changes in the nonsense transcripts in cells, which can not be translated to the protein at the first place.

### The LOXL4 level was negatively associated with the OS pathway activity in the content of the BCa chemoresistance

**Figure 5:**
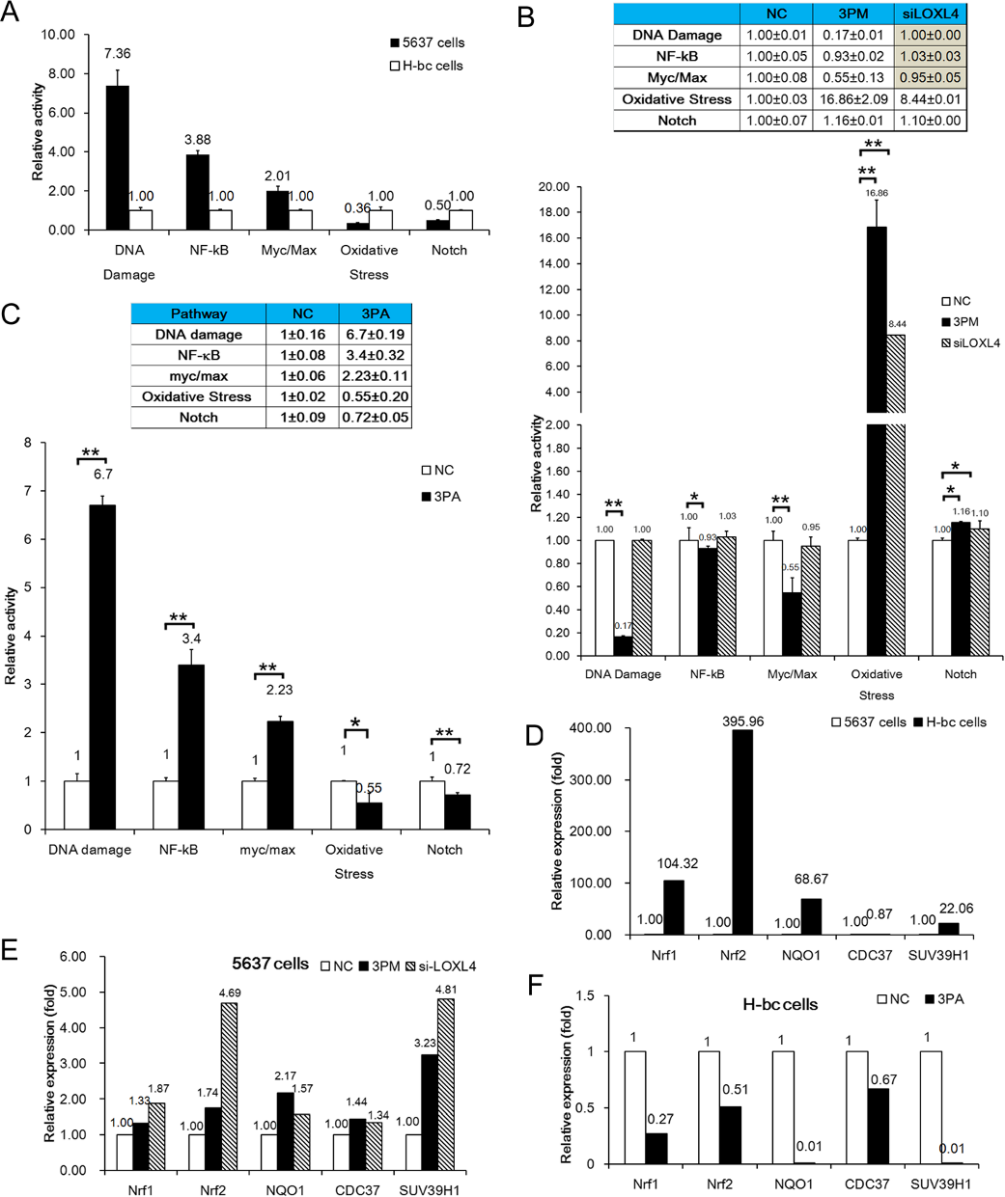
**The effects of the forced reversal of both miR-193a-3p and LOXL4 levels on the activity of the signaling pathways in 5637 versus H-bc cells. A**, The relative activities (in mean ± S.D) of five indicated pathways in 5637 versus H-bc cells. **B**, The relative pathway activities in the LOXL4 siRNA or miR-193a-3p mimic (3 PM) versus the NC transfected 5637 cells. **C**, The relative pathway activities in the miR-193a-3p antagomiR (3PA) versus the NC transfected H-bc cells. **D**, The level of the OS pathway related mRNAs in 5637 and H-bc cells. **E**, The level of the OS pathway related mRNAs in the miR-193a-3p mimic or siRNA versus the NC transfected 5637 cell. **F**, The level of the OS pathway related mRNA s in the miR-193a-3p antagomiR versus the NC transfected H-bc cells. (*, p <0.05; **,P < 0.01).

For the mechanistic insights, we used the Qiagen™ pathway reporter assay to compare the activities of the following five chemoresistance associated signaling pathways: DNA damage, Notch, NF-κB, Myc/Max and Oxidative Stress (OS) pathways in 5637 versus H-bc cells. The activities of the following three pathways: DNA damage, NF-κB and myc/max pathways were higher by no less than 2 folds in 5637 than H-bc cells (Figure 5A. The activities of these three pathways were reduced in the miR-193a-3p mimic transfected 5637 (Figure 5B) and elevated in the antagomiR transfected H-bc cells (Figure 5C), indicating a negative association of which with the BCa chemoresistance. The opposite was true for both OS and Notch pathways (Figure 5A-C). We then compared the pathway activities in the LOXL4 siRNAs versus the mock siRNA transfected 5637 cells. Although no or marginal effect on the activities of the DNA damage, NF-κB and Myc/Max pathways was observed, both OS and Notch pathways were activated in the LOXL4 siRNA transfected to a similar extent observed in the mimic transfected 5637 cells (Figure 5B) with the OS pathway’s response most drastically, Therefore, the LOXL4’s role to relay the miR-193a-3p’s effect on the BCa chemoresistance is principally accomplished *via* its effect on the OS pathway. We further measured the mRNA levels of the genes encoding two master transcription factors in OS pathway: Nrf1 and Nrf2, one downstream gene of OS pathway: NQO1 [32], and two LOXL4 interaction genes: CDC37 [33] and SUV39H1 [34] by qRT-PCR analysis. Coincidently, the Nrf1, Nrf2, NQO1 and SUV39H1 mRNAs were higher in H-bc than 5637 cells (Figure 5D), and were raised in the miR-193a-3p mimic and LOXL4 siRNA transfected 5637 cells (Figure 5E), while repressed in the miR-193a-3p antagomiR transfected H-bc cells (Figure 5F). Although being slightly lower in H-bc than 5637 cells, the CDC37 level in 5637 cells was also significantly raised by the LOXL4 siRNA transfection. In conclusion, there is a very strong biochemical and biological link between the LOXL4 level and the OS pathway activity in the content of the BCa chemoresistance.

### miR-193a-3p promotes Pa chemoresistance of BCa *via*repressing both SRSF2 and LOXL4 expression in BCa tumor xenografts in nude mice

**Figure 6:**
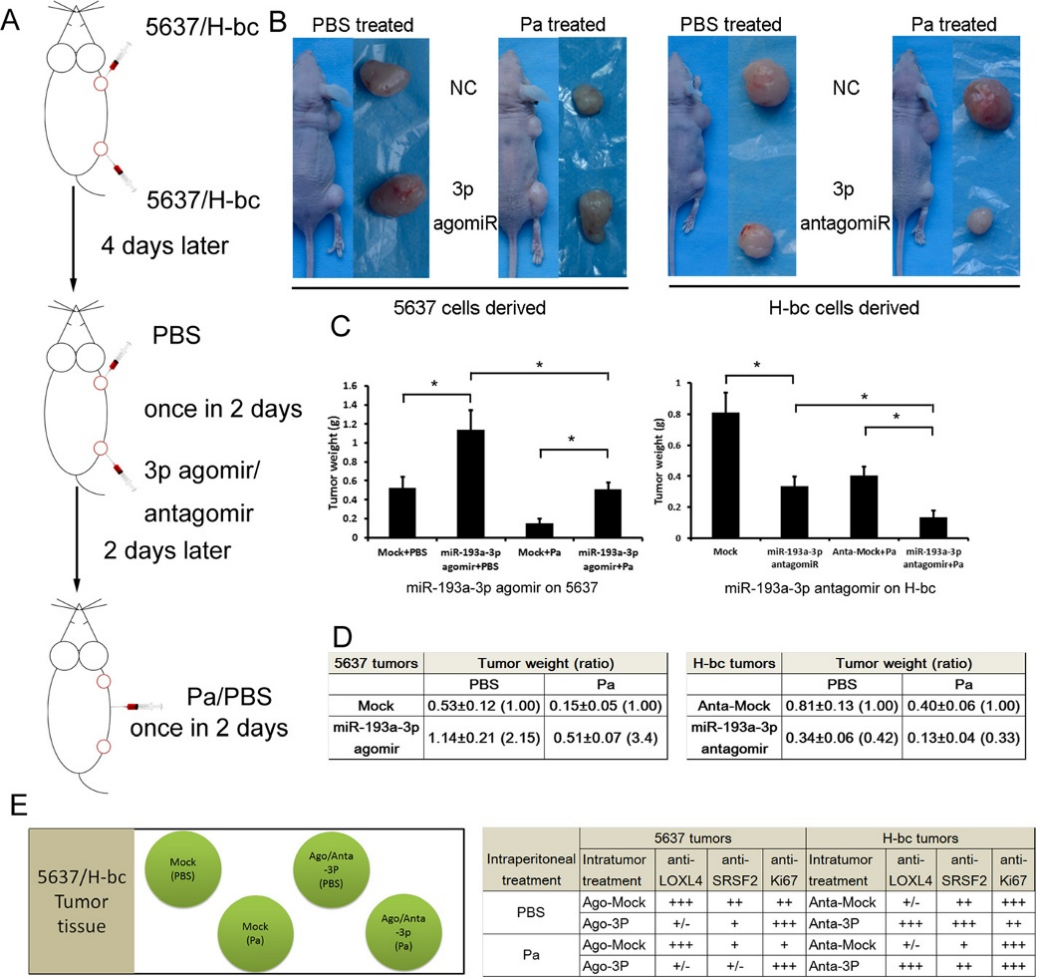
**The miR-193a-3p’s effect on both the** ***in vivo*** **growth and Pa chemoresistance of 5637 and H-bc derived xenografts in nude mice. A**, The experimental scheme, 5637 cells or H-bc cells were subcutaneously injected at four points at back of each nude mice, respectively. From the day 4^th^, PBS, the miR-193a-3p agomiR/antagomiR or the scramble sequence control (Mock) was intratumourly injected into a pair of 5637/H-bc tumors on right back site (NB, the other pair of tumor was injected with the reagents that has nor relevance in this study), respectively, once every two days for 5 times as indicated. From day 6^th^, each of three mice for 5637 or H-bc received the intraperitoneal injection of PBS or Pa once every two days for 5 times, respectively. **B**, The image of the representative mice with tumors on the day 25^th^. **C**, The mean ± SD of tumor weight of the tumor from same treatment was calculated and plotted, *, p < 0.05, and summarized in table **D** **E**, The 5637 or H-bc tumor tissues from each group were fixed on the same slide and immunostained with the indicated antibodies. Levels of Ki67, SRSF2, and LOXL4 proteins in each indicated tumor tissues were scored and summarized.

To minimize the inter-mouse bias, 5637 (1.5 × 10^7^ cells/site) or H-bc (0.7 × 10^7^ cells/site) cells were subcutaneously injected at two back sites of six mice each. An intratumor injection of miR-193a-3p agomiR/antagomiR into the 5637/H-bc derived tumors on the left back of mice was initiated on the 4^th^ day and repeated four times once in two days. The intraperitoneal injection of PBS or Pa was started on day 6^th^ into three mice each in either 5637 or H-bc groups and repeated four times once in two days (Figure 6A). The tumor mass was weighed on day 25^th^. With half less cells injected, the H-bc derived tumors were significantly heavier than 5637 derived (0.81 g/0.53 g = 1.53 in tumor weight) (Figure 6C and D), suggesting a miR-193a’s promoting role in the *in vivo* tumor growth. This conclusion was supported by the experiments where both 5637 and H-bc tumor xenografts were established in the same mice (not show). An intratumor injection of miR-193a-3p agomiR in comparison with the mock into the 5637 cell derived tumor xenograft resulted in a bigger tumor mass of 5637 cell derived tumor xenografts (0.81/0.53 = 1.53). The reverse was true from the compatible experiments with the antagomiR in H-bc tumor mice (0.13/0.40 = 0.33 in tumor weight). Therefore, the miR-193a-3p is capable to promote the *in vivo* BCa tumor growth in nude mice. In a full agreement with the observation that H-bc was more Pa-resistant than 5637 cells in cultured cells (Figure 1), an intraperitoneal injection of Pa reduced the 5637 tumors more dramatically than the H-bc tumor: the tumor weight ratio of the Pa treated over the PBS control is 0.283 (5637) versus 0.40 (H-bc) (Figure 6D).

We further assayed the levels of SRSF2, LOXL4 and Ki67 (an indicator for cell proliferation) in the tumor sessions of the Pa-treated versus PBS-treated mice (Figure 6E and Additional file 1: Figure S1), by the immuno-histological analysis. The intratumor injection of either miR-193a-3p’s agomiR (into 5637)/antagomiR (into H-bc tumor) indeed led the expected changes of both SRSF2 and LOXL4 proteins in tumor tissues (Figure 6E), and consolidate the conclusion that miR-193a-3p promotes both the growth and chemoresistance of the BCa cell derived tumor xenografts in nude mice.

## Discussion

In our previous study, we showed that SRSF2, as a key executor of the miR-193a-3p’s impact on the 5-FU resistance in HCC [29]. In the present work, LOXL4 as another direct target of miR-193a-3p has been shown having an active role in an implementation of the miR-193a-3p’s positive impact on the BCa chemoresistance (Figures 1, 2 and 3) to great extent. We have shown that LOXL4 mediated the miR-193a-3p’s chemoresistance effects to four drugs, but not the Ad’s (Figure 4A). The drug specific chemoresistance profile mediated by SRSF2 differs with LOXL4 by that the only drug not affected is EH instead (Figure 4A). The synergistic action by these two genes was only seen in the case of the chemoresistance to EH and Ad, respectively (Figure 4A). Therefore, the miR-193a-3p’s impact on the BCa chemoresistance is significantly achieved by its targeting repression of both LOXL4 and SRSF2 expression.

The LOX family proteins, including LOXL4 are required for a collagen remodeling at the metastatic site of cancer [35–37]. Expression of LOXL4 can be activated by hypoxia and is positively associated with the invasive/metastatic state of breast cancer cells [38]. Its overexpression has been suggested as a diagnostic biomarker [35, 36, 39] and even a therapeutic target for head and neck cancer treatment [40]. On the other hand, the tumor suppressor role of LOXL4 was also reported in colorectal adenocarcinomas [41]. The disparate state of the LOXL4 role in carcinogenesis has been attributed to the aberrant regulation at the level of the alternative mRNA splicing, the products of which possess either pro- or anti-oncogenic functions [37]. The silenced state of the LOXL4 gene by DNA methylation was previously reported in BCa cells [42]. However, a methyl-capture seq based methylomic analysis (not shown) showed that the LOXL4 gene is hypomethylated in 5637, H-bc and UM-UC-3 cell lines, disregard of the drastic contrast in its expression (Figure 1). Therefore, the miR-193a-3p mediated regulation at the post-transcriptional levels likely has a predominant role in the LOXL4 expression in BCa multi-chemoresistance (Figure 3A and B).

OS pathway is an important cell defense and survival pathway. It protects cancer cells from diverse hostile stimuli, including chemotherapeutics by activating the transcription of a number of cytoprotective genes [43]. Cancer cells with an elevated level of Nrf2 (the key transcriptional factor of OS pathway) are more resistant to various chemotherapeutic drugs, including etoposide, carboplatin, cisplatin, 5-fluorouracil, and doxorubicin [43]. Besides, ectopic expression of Nrf2 in the low expressing cancer cell lines renders the cells a resistance to a variety of anti-cancer agents, whereas siRNA-mediated inhibition of Nrf2 in cells with high expression levels of Nrf2 sensitized cell to the drug cytotoxicity [43]. Indeed, the chemoresistant H-bc cells have a significantly higher level of both Nrf1 and Nrf2 (Figure 5D) and a higher OS pathway activity than the chemosensitive 5637 cells (Figure 5A). Importantly, we showed for the first time that miR-193a-3p can positively regulates the OS pathway, most likely achieved by its repressing effect on the LOXL4 expression (Figure 5B)

**Figure 7:**
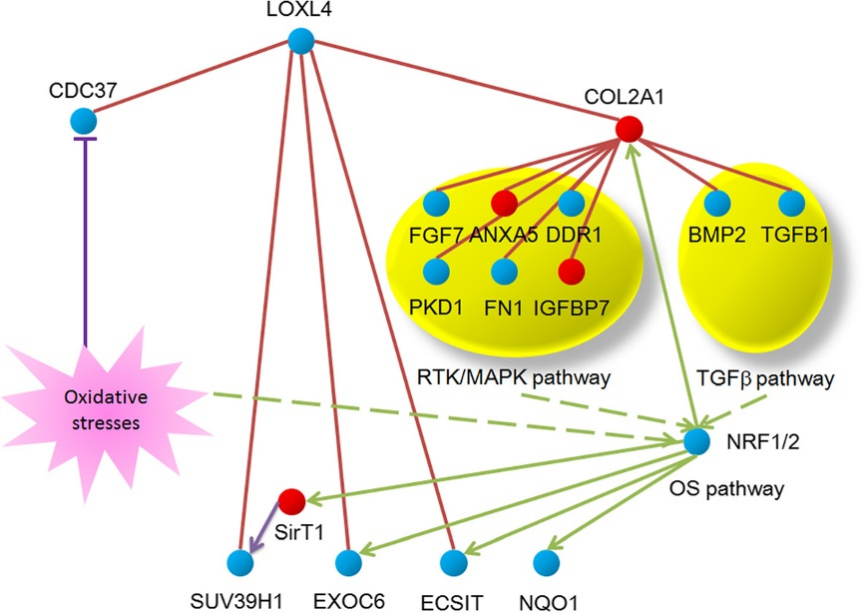
**A Simplified interaction map between the LOXL4 protein with the OS pathway by both protein-protein interaction analysis and literature mining.** The red solid lines specify a direct physical interaction between the LOXL4 protein and the indicated proteins. The green arrow lines indicate transcriptional activation by the OS pathway. The green dashed arrow-lines signify the impact on OS pathway via effect on the phosphorylation state, activity or the nuclear-cytoplasmic distribution of Nrf1/2 proteins. T headed purple line indicates the direct interaction of SirT1 with SUV39H1 with a repression effect. The red circles designate the genes that expressed higher in H-bc than 5637 cells, while the blue circles indicate the genes in a reverse state of expression (based on the RNA-seq data, not shown in this paper).

A network analysis to link the LOXL4 gene to the OS pathway was performed by combining the literature mining with an analysis of the public protein-protein interaction databases: **Wiki-PI** [http://severus.dbmi.pitt.edu/wiki-pi/] and **BioGRID 3.2** [http://thebiogrid.org/]. Among 15 proteins that directly interact with LOXL4 protein, three have a reported OS pathway association. SUV39H1 is a histone methyl transferase, responsible for the histone H3K9 bi-and tri-methylation [34]. It actively engages in formation of the transcriptional silenced heterochromatin region in genome. SUV39H1 is stabilized by SirT1, the transcription of which is under regulation of OS pathway [34] and therefore, maintains the genome integrity in the stressed cells. CDC37 is a molecular chaperone that form complex with Hsp90 and a variety of protein kinases. It is thought to play a critical role in directing Hsp90 to its target kinases, and this interaction is destabilized by oxidative stress stimuli for the cellular apoptosis to occur [33]. ECSIT is an adapter protein of the Toll-like and IL-1 receptor signaling pathway that is involved in multiple signal pathways, such as NF-κB and BMP. It may act as a sensor to protect cells by activation of protective molecular mechanisms in response to Oxidative stresses [44]. Interestingly, the following two LOXL4 interacting proteins: EXOC6 and COL2A1 genes are the direct target of the OS pathway suggested by a Nrf2 centered network analysis: **NRF2omic database** [http://nrf2.elte.hu] [45, 46]. The EXOC6 gene encodes a member of SEC15 family contributing to the docking of exocytic vesicles with fusion sites on the plasma membrane [47]. COL2A1 encodes the alpha-1 chain of type II collagen [48], directly interact with the following 8 proteins: 6 components of RTK/MAPK pathway (ANXA5, DDR1, FGF7, PKD1, FN1 and IGFBP7) and 2 components (BMP2 and TGFB1) in the TGF-beta pathway. The phosphorylation state and activity of Nrf2 are under the control of both RTK/MAPK and TGF-beta pathways [49]. Indeed, along with a rise of miR-193a-3p level and reduction of the LOXL4 level in 5637 cells, the drug-triggered cell death was rescued (Figure 5). However, we found no evidence for that the force reversal of both miR-193-3p and SRSF2/LOXL4 levels in 5637 and H-bc cells have a detectable effect on the level of Nrf2 and its phosphorylated form as well as its nuclear-cytoplasmic distribution (data not shown). Therefore, the effect on the OS pathway (Figure 5) by miR-193a-3p *via* repression of LOXL4 seems to be produced by other unknown mechanism(s). The direct interaction between the LOXL4 protein and the master transcription factors Nrf1 and Nrf2 in OS pathway has not been reported so far. However five LOXL4 integrating protein partners discussed above may act as the key components of the OS pathway based cellular response to the damage signals, such as the chemotherapeutic drugs (Figure 7). It is clear that further efforts are needed to address how the miR-193a-3p affected the OS pathway through its repression of the LOXL4 expression in the content of the BCa chemoresistance.

## Conclusion

In this study, we demonstrated that miR-193a-3p promotes the multi-chemoresistance of BCa *via* repressing of the LOXL4 expression and therefore activating the OS pathway. This study also provided a new set of genes in this newly identified miR-193a-3p/LOXL4/Oxidative Stress axis as the diagnostic targets for the guided anti-bladder cancer chemotherapy, including the level of the miR-193a-3p gene, both LOXL4 and SRSF2 gene and the key OS pathway associated genes in both cancer tissues and urine sediments.

## Methods

### Cell lines and culture

Five bladder cancer cell lines were purchased from the Chinese Academy of Cell Resource Center (Shanghai, China): 5637 (ATCC NO. HTB-9), T24(ATCC NO. HTB-4), UM-UC-3 (ATCC NO. CRL-1749), Biu87 (established by department of Urology of Beijing Medical University in 1987 [50]) and H-bc cell lines (established by cancer research Institute of Kunming Medical College, 1986). UM-UC-3 cells are cultured in MEM plus 10% Fetal Bovine serum, and the other cell lines are cultured in RPMI1640 (Invitrogen, USA) + 10% Fetal Bovine serum (Invitrogen, USA) and 1% glutamine at 37°C in 5% CO_2_.

### Chemotherapeutics

The clinic grade of drugs are used (NCI Dictionary of Cancer Terms, http://www.cancer.gov/dictionary), Pirarubicin (Pi, Wanle, Shenzhen), Paclitaxel (Pa, Taiji, Sichuan), Adriamycin (Ad, Pfizer, Jiangsu), Epirubicin Hydrochloride (EH, Haizheng, Zhejiang), and Cisplatin (Ci, Haosen, Jiangsu).

### Chemoresistance profiling (IC_50_ determination)

Cells at the logarithmic phase of growth were seeded in triplicate in 96-well plates at a density of 0.5 × 10^4^/well and treated with 4 fold serious diluted drugs for 72 hours. The cell survival was then measured by a thiazolyl blue tetrazolium bromide (MTT, 490 nm reading)-based cell viability assay [51]. Both the linear regression parameters and the IC_50_ (the concentration of drug required for 50% cells killed) with the no-drug control as the reference were calculated. The relative chemoresistance was presented as the fold for the IC_50_ of the cell lines over the lowest IC_50_.

### The reagents for the transient transfection and *in vivo*assays

All the mimic, agomiR, antagomiR, siRNA, the scramble sequence (negative control, NC) and the riboFECT CP transfection kit were supplied by Ribobio, Guangzhou, China. Transfection of both ribonucleic acid reagents mentioned above and the reporter plasmids was performed according to the manufactory’s instruction. Chemically modified mimic oligonucleotides (agomir) were synthesized to regulate miR-193a-3p/5p expression *in vivo*. The 3’ end of the oligonucleotides was conjugated to cholesterol, and all the bases were 2’-OMe modified. The agomir oligonucleotides were deprotected, desalted and purified by high-performance liquid chromatography.

### The luciferase reporter assay

A full length of the human LOXL4 3’-untranslated region (1325 bp) with a wide type and mutant target sequence for miR-193a-3p were cloned into 3’ flank of luciferase coding sequence of pGL3 (Invitrogen, Carlsbad, CA, USA) to construct pGL3-luc-LOXL4 WT and pGL3-luc-LOXL4 Mut, respectively. All the constructs were confirmed by DNA sequencing. Cells were seeded into 96-well plates at around 1 × 10^4^ cells per well and transfected with a mixture of 50 ng pGL3-luc-LOXL4 WT or Mut, 5 ng Renilla plus 5 pmol mimic or NC nucleotides, with the riboFECT CP transfection kit according to the manufacturer’s instruction. Both firefly and Renilla luciferase activities were measured around 18 hours after transfection by the Dual-Luciferase Reporter Assay System (Promega, Madison, Wisconsin, USA) using a Promega GloMax 20/20 luminometer. The relative firefly luciferase activities were normalized with the Renilla luciferase activities, which served as an internal control for transfection efficiency for the standard analysis.

### The signaling pathway analysis

The following five signaling pathway reporter constructs were obtained from Qiagen (Hilden, German). DNA damage, Notch, NF-κB, Myc/Max and Oxidative Stress (OS) pathways and analyzed according to the manufacturer’s instruction. Briefly, the cells were transfected in triplet with each firefly luciferase reporter construct in combination with the Renilla luciferase based control construct using the riboFECT CP transfection reagent, and both luciferase activities in cell extracts at 18 hours after transfection were measured. The relative luciferase activities (luciferase unit) of the pathway reporter over the negative control in the transfected cells were calculated as a measurement of the pathway activity.

### Apoptosis analysis

Cells were harvested and rinsed with PBS twice. Then 5 μl of FITC-labeled enhanced-annexinV and 5 μl (20 μg/ml) of propidium iodide were added into 100 μl cell suspension. Upon incubation in the dark for 15 min at room temperature, samples were diluted with 400 μl PBS. Flow cytometry was carried out on a FACS calibur instrument. The result was analyzed according to the manufacturer’s instruction. The experiments were performed independently three times and a representative was shown.

### RNA analysis

Total RNA was isolated from the cells at the logarithmic phase by Trizol technology (Tiangen Biotech Co., Ltd., Beijing, China). For mRNA analysis, the cDNA primed by oligo-dT was made with a prime Script RT reagent kit (Tiangen Biotech Co., Ltd., Beijing, China) and the mRNA level of the genes LOXL4, SRSF2, Nrf-1,Nrf-2, CDC37, SUV39H1, and NQO1 were quantified by a duplex-qRT-PCR analysis where the Taqman probes in a different fluorescence for the β-actin (provided by Shing Gene, Shanghai, China) was used in the FTC-3000P PCR instrument (FUNGLYN BIOTECH INC, Toronto, Canada). Using the 2^-ΔΔ^ Ct method, the normalization with the β-actin level was performed before the relative level of the target genes was compared. The sequences of primers and probes used for the qRT-PCR analysis are:

hLOXL4F: 5′-TATGGCAGAAGGAGACCCG-3′

hLOXL4R: 5′-CCATTCCTCACTGAACCCG-3′

hLOXL4 probe: 5′-CY5-CATTCTCTGCAACAGTGTCTTGTGACTCC-3′

hSRSF2F: 5′-GTGCAAATGGCGCGCTAC-3′

hSRSF2R: 5′-CTGGAACGGCTCCGACTC-3′

hSRSF2 probe: 5′rox-ACCGCCACCCCGCAGGTACG-3′

hNrf1F: 5′-CTTCAGAATTGCCAACCACG-3′

hNrf1R: 5′-TGCTTGCGTCGTCTGGATG-3′

hNrf1 probe: 5′FAM-CCGTGGCTGATGGAGAGGTGGAAC-3′

hNrf-2 F: 5′-AGCCCCTGTTGATTTAGACGG-3′

hNrf-2R: 5′-TGGCTTCTGGACTTGGAACC-3′

hNrf-2 probe: 5′cy5-CAAGTTTGGGAGGAGCTATTATCCATTCC-3′

hCDC37F: 5′-GACAATCGTCATGCAATTTATCC-3′

hCDC37R: 5′-CCCTCCATGTACTGGCGATC-3′

hCDC37 probe: 5′cy5-CCTGCTTCCGGCAGTTCTTCACTAAG-3′

hSUV39H1F: 5′-AAGTCGAGTACCTGTGCGATTAC-3′

hSUV39H1R: 5′-AAGTCCTTGTGGAACTGCTTGA-3′

hSUV39H1 probe: 5′cy5-CGAACAGGAATATTACCTGGTGAAATGGC-3′

hNQO1F: 5′-AACTTCAATCCCATCATTTCCA-3′

hNQO1R: 5′-TTTATAAGCCAGAACAGACTCGG-3′

hNQO1 probe: 5′cy5-CTGAAGGACCCTGCGAACTTTCAGTATC-3′

hACTBF: 5′-GCCCATCTACGAGGGGTATG-3′

hACTBR: 5′-GAGGTAGTCAGTCAGGTCCCG-3′

hACTB probe: 5′HEX-CCCCCATGCCATCCTGCGTC-3′

### Bulge-Loop™ miRNA qRT-PCR

For detecting and quantifying the expression of specific miRNAs, RNA was reverse transcribed using Bulge-Loop™ miRNA qRT-PCR Primer Set (Ribobio) and quantified by the SYBR Green-based real-time PCR analysis in the FTC-3000P (FUNGLYN BIOTECH INC, Canada). The Ct values of the target miRs were normalized to the Ct values of U6 RNA before quantification using the 2^-ΔΔCt^ method.

### Western blot analysis of protein

Cells were lyzed with a lysis buffer (60 mM Tris–HCl, pH6.8, 2% SDS, 20% glycerol, 0.25% bromophenol blue, 1.25% 2-mercaptoethanol) and heated at 100°C for 10 min before the electrophoresis/Western analysis. The anti-LOXL4 (AP17245b), anti-SRSF2 (AP2800a), anti-GAPDH (AM1020a), anti-rabbit IgG peroxidase-conjugated antibody(LP1001b), and HRP goat anti-mouse IgG antibody (LP1002a) were provided by Wuxiphama, Shanghai, China. The target bands were revealed by an enhanced chemiluminescence reaction (Thermo Fisher Scientific. Waltham, MA, USA) and the relative density (level) of proteins over the GAPDH band were quantified with the Gel-Pro Analyzer (Media Cybernetics, Rockville, MD, USA).

### The *in vivo*studies

Animal experiments were undertaken in accordance with the National Institutes of Health Guide for the Care and Use of Laboratory Animals. BALB/c male nude mice of 8–12 weeks of age were used for this study. 5637 or H-bc cells were embedded in BD Matrigel™ Matrix (Becton, Dickinson, NJ, USA) [52] and subcutaneously injected into at four sites at back of mice as following: 1.7 × 10^7^ cells/site for 5637, 0.7 × 10^7^ cells/site for H-bc,4 sites/mouse, 6 mice for 5637, 6 mice for H-bc, respectively. From the 4^th^ day after cell injection, all 5637 generated tumors on the left back of nude mice were intratumorally injected with 2nM miR-193a-3p agomiR/Mock, while H-bc generated tumors on the left back of nude mice were injected with 4nM miR-193a-3p/Mock antagomiR. From the 6^th^ day after cell injection, 3 mice from 5637 and 3 from H-bc were intraperitoneally received Pa (45 ug/mouse) once in 2 days. The remaining 6 mice (3 from 5637 and 3 from H-bc) received phosphate-buffered saline (PBS) as a mock treatment control. Mice were humanely sacrificed on day 25, and the tumors were weighed and photographed. The tumor weight was described as the mean ± S.D.

Expressions of SRSF2, LOXL4, and Ki67 proteins were measured using immunochemical analysis on 5-mm slices of formalin fixed paraffin-embedded tumor xenografts in nude mice. To avoid inter-treatment bias, the tissue slices from all the six groups were made on a single slide and subject to the same immuno-staining simultaneously. Antigens were retrieved by pretreating dewaxed sections in a microwave oven at 750 watts for 5 min in a citrate buffer (pH 6) processed with the Super Sensitive Link-Labeled Detection System (Biogenex, Menarini, Florence, Italy). The enzymatic activities were developed using 3-amino-9-ethylcarbazole (Dako, Milan, Italy) as a chromogenic substrate. Following counter staining with Mayer hematoxylin (Invitrogen), slides were mounted in aqueous mounting medium (glycergel, Dako). Pictures were taken using LEICA DM 4000B microscope (Wetzlar, German), while the relative level of each protein was calculated using LEICA software, percentage of the mock over the chemotherapeutic treated tumors was calculated and plotted.

### Statistical analysis

Data are presented as means, and error bars indicate the S.D. or S.E. All statistical analyses were performed with Excel (Microsoft, Redmond, WA) or Prism (GraphPad Software Inc., La Jolla, CA). Two-tailed Student’s *t*-test, a one-way analysis of variance or Mann–Whitney *U* test was used to calculate statistical significance. A p-value of <0.05 was considered to be significant.

## Electronic supplementary material

Additional file 1: Figure S1: Immunostaining analysis of tumor tissues from *in vivo* study. **A**, The 5637 and H-bc tumor tissues from each group were fixed on one slide and immunostained for indicated antibody, respectively. Levels of Ki67, SRSF2, and LOXL4 proteins in each were determined by immunostaining. **B**, Levels of Ki67, SRSF2, and LOXL4 proteins were summarized in the table. (PDF 542 KB)

## Abbreviations

BCa: Bladder cancer
MiR: MicroRNA
HCC: Hepatocellular cancer
NSCLC: Non-small cell lung cancer
ROS: Reactive oxygen species
OS: Oxidative stress
Pi: Pirarubicin
Pa: Paclitaxel
Ad: Adriamycin
EH: Epirubicin hydrochloride
Ci: Cisplatin.

## References

[1] Burger M, Catto JW, Dalbagni G, Grossman HB, Herr H, Karakiewicz P, Kassouf W, Kiemeney LA, La Vecchia C, Shariat S (2013). Epidemiology and risk factors of urothelial bladder cancer. Eur Urol.

[2] von der Maase H, Sengelov L, Roberts JT, Ricci S, Dogliotti L, Oliver T, Moore MJ, Zimmermann A, Arning M (2005). Long-term survival results of a randomized trial comparing gemcitabine plus cisplatin, with methotrexate, vinblastine, doxorubicin, plus cisplatin in patients with bladder cancer. J Clin Oncol.

[3] Chang JS, Lara PN, Pan C-X (2012). Progress in personalizing chemotherapy for bladder cancer. Adv Urol.

[4] Bartel DP (2009). MicroRNAs: target recognition and regulatory functions. Cell.

[5] Di Leva G, Garofalo M, Croce CM (2014). MicroRNAs in cancer. Annu Rev Pathol Mech Dis.

[6] Cheng CJ, Slack FJ (2012). The duality of oncomiR addiction in the maintenance and treatment of cancer. Cancer J (Sudbury, Mass).

[7] Blandino G, Fazi F, Donzelli S, Kedmi M, Sas-Chen A, Muti P, Strano S, Yarden Y (2014). Tumor suppressor microRNAs: a novel non-coding alliance against cancer. FEBS Lett.

[8] Ling H, Fabbri M, Calin GA (2013). MicroRNAs and other non-coding RNAs as targets for anticancer drug development. Nat Rev Drug Discov.

[9] Sethi S, Ali S, Philip PA, Sarkar FH (2013). Clinical advances in molecular biomarkers for cancer diagnosis and therapy. Int J Mol Sci.

[10] Haenisch S, Cascorbi I (2012). miRNAs as mediators of drug resistance. Epigenomics.

[11] Su S, Chang Y, Andreu-Vieyra C, Fang J, Yang Z, Han B, Lee A, Liang G (2013). miR-30d, miR-181a and miR-199a-5p cooperatively suppress the endoplasmic reticulum chaperone and signaling regulator GRP78 in cancer. Oncogene.

[12] K-i K, Imoto I, Mogi S, Omura K, Inazawa J (2008). Exploration of tumor-suppressive microRNAs silenced by DNA hypermethylation in oral cancer. Cancer Res.

[13] Heller G, Weinzierl M, Noll C, Babinsky V, Ziegler B, Altenberger C, Minichsdorfer C, Lang G, Döme B, End-Pfützenreuter A (2012). Genome-wide miRNA expression profiling identifies miR-9-3 and miR-193a as targets for DNA Methylation in non–small cell lung cancers. Clin Cancer Res.

[14] Avci CB, Harman E, Dodurga Y, Susluer SY, Gunduz C (2013). Therapeutic potential of an anti-diabetic drug, metformin: alteration of miRNA expression in prostate cancer cells. Asian Pac J Cancer Prev.

[15] Tahiri A, Leivonen SK, Luders T, Steinfeld I, Ragle Aure M, Geisler J, Makela R, Nord S, Riis ML, Yakhini Z, Kleivi Sahlberg K, Børresen-Dale AL, Perälä M, Bukholm IR, Kristensen VN (2014). Deregulation of cancer-related miRNAs is a common event in both benign and malignant human breast tumors. Carcinogenesis.

[16] Chen D, Cabay RJ, Jin Y, Wang A, Lu Y, Shah-Khan M, Zhou X (2013). MicroRNA deregulations in head and neck squamous cell carcinomas. J Oral Maxillofac Res.

[17] Yong FL, Law CW, Wang CW (2013). Potentiality of a triple microRNA classifier: miR-193a-3p, miR-23a and miR-338-5p for early detection of colorectal cancer. BMC Cancer.

[18] Gao X, Lin J, Li Y, Gao L, Wang X, Wang W, Kang H, Yan G, Wang L, Yu L (2011). MicroRNA-193a represses c-kit expression and functions as a methylation-silenced tumor suppressor in acute myeloid leukemia. Oncogene.

[19] Li Y, Gao L, Luo X, Wang L, Gao X, Wang W, Sun J, Dou L, Li J, Xu C (2013). Epigenetic silencing of microRNA-193a contributes to leukemogenesis in t (8; 21) acute myeloid leukemia by activating the PTEN/PI3K signal pathway. Blood.

[20] Iliopoulos D, Rotem A, Struhl K (2011). Inhibition of miR-193a expression by Max and RXRα activates K-Ras and PLAU to mediate distinct aspects of cellular transformation. Cancer Res.

[21] Noh H, Hong S, Dong Z, Pan ZK, Jing Q, Huang S (2011). Impaired microRNA processing facilitates breast cancer cell invasion by upregulating urokinase-type plasminogen activator expression. Genes Cancer.

[22] Uhlmann S, Mannsperger H, Zhang JD, Horvat EÁ, Schmidt C, Küblbeck M, Henjes F, Ward A, Tschulena U, Zweig K (2012). Global microRNA level regulation of EGFR‒driven cell‒cycle protein network in breast cancer. Mol Syst Biol.

[23] Nakano H, Yamada Y, Miyazawa T, Yoshida T (2013). Gain-of-function microRNA screens identify miR-193a regulating proliferation and apoptosis in epithelial ovarian cancer cells. Int J Oncol.

[24] Salvi A, Conde I, Abeni E, Arici B, Grossi I, Specchia C, Portolani N, Barlati S, De Petro G (2013). Effects of miR-193a and sorafenib on hepatocellular carcinoma cells. Mol Cancer.

[25] Wang J, Yang B, Han L, Li X, Tao H, Zhang S, Hu Y (2013). Demethylation of miR-9-3 and miR-193a genes suppresses proliferation and promotes apoptosis in non-small cell lung cancer cell lines. Cell Physiol Biochem.

[26] Kwon JE, Kim BY, Kwak SY, Bae IH, Han YH (2013). Ionizing radiation-inducible microRNA miR-193a-3p induces apoptosis by directly targeting Mcl-1. Apoptosis.

[27] Yu T, Li J, Yan M, Liu L, Lin H, Zhao F, Sun L, Zhang Y, Cui Y, Zhang F, Li J, He X, Yao M (2014). MicroRNA-193a-3p and -5p suppress the metastasis of human non-small-cell lung cancer by downregulating the ERBB4/PIK3R3/mTOR/S6K2 signaling pathway. Oncogene.

[28] Santarpia L, Calin GA, Adam L, Ye L, Fusco A, Giunti S, Thaller C, Paladini L, Zhang X, Jimenez C (2013). A miRNA signature associated with human metastatic medullary thyroid carcinoma. Endocr Relat Cancer.

[29] Ma K, He Y, Zhang H, Fei Q, Niu D, Wang D, Ding X, Xu H, Chen X, Zhu J (2012). DNA methylation-regulated miR-193a-3p dictates resistance of hepatocellular carcinoma to 5-fluorouracil via repression of SRSF2 expression. J Biol Chem.

[30] Baek D, Villén J, Shin C, Camargo FD, Gygi SP, Bartel DP (2008). The impact of microRNAs on protein output. Nature.

[31] Zhao Y, Lin J, Xu B, Hu S, Zhang X, Wu L (2014). MicroRNA-mediated repression of nonsense mRNAs.

[32] Bloom DA, Jaiswal AK (2003). Phosphorylation of Nrf2 at Ser40 by protein kinase C in response to antioxidants leads to the release of Nrf2 from INrf2, but is not required for Nrf2 stabilization/accumulation in the nucleus and transcriptional activation of antioxidant response element-mediated NAD (P) H: quinone oxidoreductase-1 gene expression. J Biol Chem.

[33] Sarkar S, Dutta D, Samanta SK, Bhattacharya K, Pal BC, Li J, Datta K, Mandal C, Mandal C (2013). Oxidative inhibition of Hsp90 disrupts the super‒chaperone complex and attenuates pancreatic adenocarcinoma in vitro and in vivo. Int J Cancer.

[34] Bosch-Presegué L, Raurell-Vila H, Marazuela-Duque A, Kane-Goldsmith N, Valle A, Oliver J, Serrano L, Vaquero A (2011). Stabilization of Suv39H1 by SirT1 is part of oxidative stress response and ensures genome protection. Mol Cell.

[35] Scola N, Goeroegh T (2010). LOXL4 as a selective molecular marker in primary and metastatic head/neck carcinoma. Anticancer Res.

[36] Weise JB, Rudolph P, Heiser A, Kruse M-L, Hedderich J, Cordes C, Hoffmann M, Brant O, Ambrosch P, Csiszar K (2008). LOXL4 is a selectively expressed candidate diagnostic antigen in head and neck cancer. Eur J Cancer.

[37] Sebban S, Golan-Gerstl R, Karni R, Vaksman O, Davidson B, Reich R (2013). Alternatively spliced lysyl oxidase-like 4 isoforms have a pro-metastatic role in cancer. Clin Exp Metastasis.

[38] Kirschmann DA, Seftor EA, Fong SF, Nieva DR, Sullivan CM, Edwards EM, Sommer P, Csiszar K, Hendrix MJ (2002). A molecular role for lysyl oxidase in breast cancer invasion. Cancer Res.

[39] Görögh T, Weise J, Holtmeier C, Rudolph P, Hedderich J, Gottschlich S, Hoffmann M, Ambrosch P, Csiszar K (2007). Selective upregulation and amplification of the lysyl oxidase like‒4 (LOXL4) gene in head and neck squamous cell carcinoma. J Pathol.

[40] Weise JB, Csiszar K, Gottschlich S, Hoffmann M, Schmidt A, Weingartz U, Adamzik I, Heiser A, Kabelitz D, Ambrosch P (2008). Vaccination strategy to target lysyl oxidase-like 4 in dendritic cell based immunotherapy for head and neck cancer. Int J Oncol.

[41] Kim Y, Roh S, Park J-Y, Kim Y, Cho DH, Kim JC (2009). Differential expression of the LOX family genes in human colorectal adenocarcinomas. Oncol Rep.

[42] Wu G, Guo Z, Chang X, Kim MS, Nagpal JK, Liu J, Maki JM, Kivirikko KI, Ethier SP, Trink B (2007). LOXL1 and LOXL4 are epigenetically silenced and can inhibit ras/extracellular signal-regulated kinase signaling pathway in human bladder cancer. Cancer Res.

[43] Jaramillo MC, Zhang DD (2013). The emerging role of the Nrf2–Keap1 signaling pathway in cancer. Genes Dev.

[44] Soler‒López M, Badiola N, Zanzoni A, Aloy P (2012). Towards Alzheimer’s root cause: ECSIT as an integrating hub between oxidative stress, inflammation and mitochondrial dysfunction. Bioessays.

[45] Türei D, Papp D, Fazekas D, Földvári-Nagy L, Módos D, Lenti K, Csermely P, Korcsmáros T (2013). NRF2-ome: an integrated web resource to discover protein interaction and regulatory networks of NRF2. Oxid Med Cell Longev.

[46] Papp D, Lenti K, Módos D, Fazekas D, Dúl Z, Türei D, Földvári-Nagy L, Nussinov R, Csermely P, Korcsmáros T (2012). The NRF2-related interactome and regulome contain multifunctional proteins and fine-tuned autoregulatory loops. FEBS Lett.

[47] Malhotra D, Portales-Casamar E, Singh A, Srivastava S, Arenillas D, Happel C, Shyr C, Wakabayashi N, Kensler TW, Wasserman WW (2010). Global mapping of binding sites for Nrf2 identifies novel targets in cell survival response through ChIP-Seq profiling and network analysis. Nucleic Acids Res.

[48] Sandelin A, Alkema W, Engström P, Wasserman WW, Lenhard B (2004). JASPAR: an open‒access database for eukaryotic transcription factor binding profiles. Nucleic Acids Res.

[49] Gañán-Gómez I, Wei Y, Yang H, Boyano-Adánez MC, García-Manero G (2013). Oncogenic functions of the transcription factor Nrf2. Free Radic Biol Med.

[50] Xu B, He Y, Wu X, Luo C, Liu A, Zhang J (2012). Exploration of the correlations between interferon-gamma in patient serum and HEPACAM in bladder transitional cell carcinoma, and the interferon-gamma mechanism Inhibiting BIU-87 proliferation. J Urol.

[51] Andrisano V, Bartolini M, Gotti R, Cavrini V, Felix G (2001). Determination of inhibitors’ potency (IC50) by a direct high-performance liquid chromatographic method on an immobilised acetylcholinesterase column. J Chromatogr B Biomed Sci Appl.

[52] Ohashi K, Marion PL, Nakai H, Meuse L, Cullen JM, Bordier BB, Schwall R, Greenberg HB, Glenn JS, Kay MA (2000). Sustained survival of human hepatocytes in mice: a model for in vivo infection with human hepatitis B and hepatitis delta viruses. Nat Med.

