# Supplementary material for: miR-193a-3p regulates the multi-drug resistance of bladder cancer by targeting the LOXL4 gene and the Oxidative Stress pathway
Source: Mol Cancer. 2014 Oct 14;13:234. doi: 10.1186/1476-4598-13-234 (PMC4200202; doi:10.1186/1476-4598-13-234)
Supplement: Supplementary file 1 — Additional file 1: Figure S1: Immunostaining analysis of tumor tissues from in vivo study. A, The 5637 and H-bc tumor tissues from each group were fixed on one slide and immunostained for indicated antibody, respectively. Levels of Ki67, SRSF2, and LOXL4 proteins in each were determined by immunostaining. B, Levels of Ki67, SRSF2, and LOXL4 proteins were summarized in the table. (PDF 542 KB) [file 12943_2014_1433_MOESM1_ESM.pdf]

Supplementary figure S1

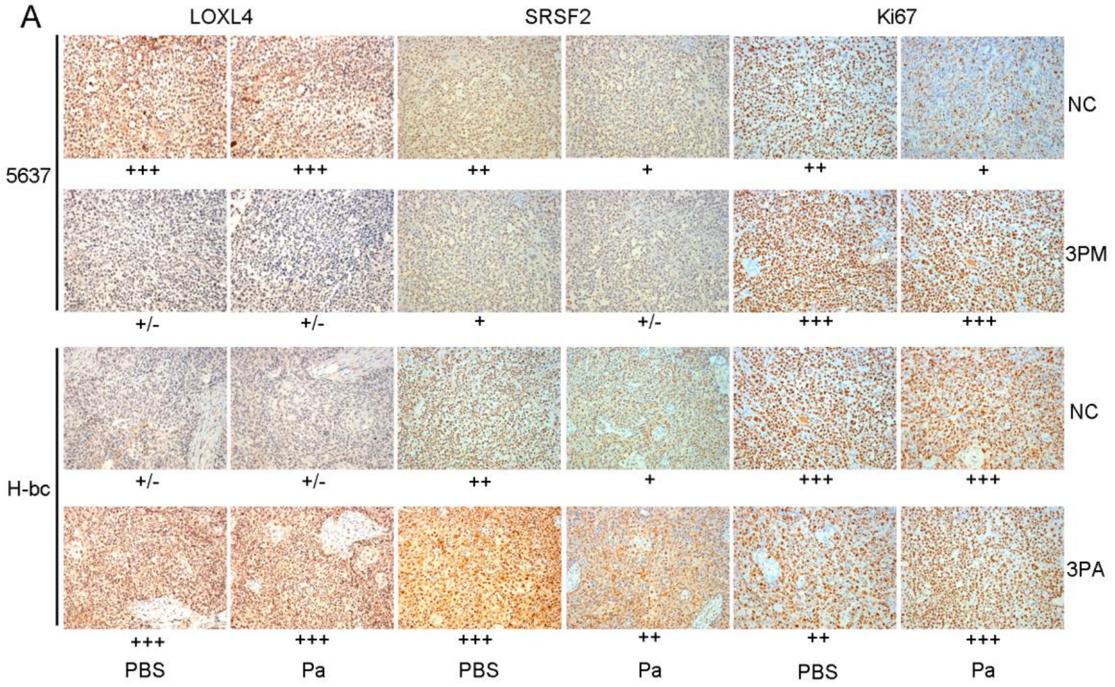

B

Intraperitoneal treatment	5637 tumors				H-bc tumors			
	Intratumor treatment	anti-LOXL4	anti-SRSF2	anti-Ki67	Intratumor treatment	anti-LOXL4	anti-SRSF2	anti-Ki67
PBS	Ago-Mock	+++	++	++	Anta-Mock	+/-	++	+++
	Ago-3P	+/-	+	+++	Anta-3P	+++	+++	++
Pa	Ago-Mock	+++	+	+	Anta-Mock	+/-	+	+++
	Ago-3P	+/-	+/-	+++	Anta-3P	+++	++	+++

Figure S1: Immunostaining analysis of tumor tissues from *in vivo* study. **A**, The 5637 and H-bc tumor tissues from each group were fixed on one slide and immunostained for indicated antibody, respectively. Levels of Ki67, SRSF2, and LOXL4 proteins in each were determined by immunostaining. **B**, Levels of Ki67, SRSF2, and LOXL4 proteins were summarized in the table.
